# The lubricating role of water in the shuttling of rotaxanes†
†Electronic supplementary information (ESI) available: Methods, structure of the rotaxane, free-energy calculation characterizing the isomerization of the ring-like molecule, free-energy landscape for the translation and conformational change of the ring in the rotaxane in vacuum, representative three-dimensional arrangements of the rotaxane in vacuum, rotation of the macrocycle along with translation, rotation of the terminal group of the dumbbell-like molecule accompanied with other movements in the rotaxane, one-dimensional free-energy decomposition, committor analysis, hydrogen bond analysis, boat–boat transformation of the macrocycle during the shuttling, solvent-accessible surface area (SASA) of the chain-like molecule along the transition coordinate in different solvents. Simulation parameters. See DOI: 10.1039/c7sc01593c



**DOI:** 10.1039/c7sc01593c

**Published:** 2017-05-16

**Authors:** Haohao Fu, Xueguang Shao, Christophe Chipot, Wensheng Cai

**Affiliations:** a Research Center for Analytical Sciences , College of Chemistry , Tianjin Key Laboratory of Biosensing and Molecular Recognition , Nankai University , Tianjin 300071 , China . Email: wscai@nankai.edu.cn; b Collaborative Innovation Center of Chemical Science and Engineering (Tianjin) , Tianjin 300071 , China; c State Key Laboratory of Medicinal Chemical Biology , Nankai University , Tianjin 300071 , China; d Laboratoire International Associé Centre National de la Recherche Scientifique et University of Illinois at Urbana-Champaign , Unité Mixte de Recherche No. 7565 , Université de Lorraine , B.P. 70239 , 54506 Vandœuvre-lès-Nancy cedex , France; e Theoretical and Computational Biophysics Group , Beckman Institute , University of Illinois at Urbana-Champaign , Urbana , Illinois 61801 , USA; f Department of Physics , University of Illinois at Urbana-Champaign , 1110 West Green Street , Urbana , Illinois 61801 , USA

## Abstract

We have investigated at the atomic level amide-based rotaxanes set in motion in four different solvents, namely, ethyl ether, acetonitrile, ethanol and water. In three non-aqueous solvents, shuttling of the macrocycle between two binding sites separated by a free-energy barrier is coupled with a conformational change and rotation, driven primarily by hydrogen-bonding interactions. The mechanism that underlies the shuttling is completely altered when the non-aqueous solvent is replaced by water. In aqueous solution, hydrophobic interactions chiefly control shuttling of the rotaxane, leading to a sharp decrease of the free-energy barrier, thereby speeding up the process. The binding sites and the reaction pathway describing shuttling vary significantly in water compared with in the other three solvents. We found that the high polarity, the hydrogen-bond donor and acceptor ability, and the minimal steric hindrance of water conspire to modify the mechanism. These three physicochemical properties are also responsible for the lubrication by water. That water completely changes the mechanism underlying the shuttling of rotaxanes, is addressed for the first time in this study, and provides valuable guidelines for the *de novo* design of molecular machines.

## Abstract 2

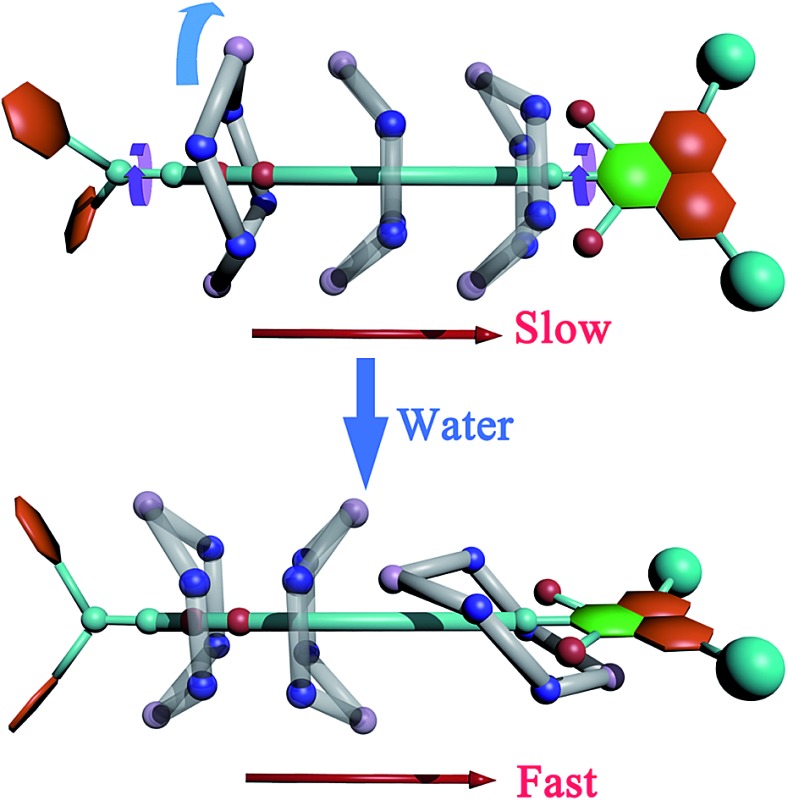
The special properties of water make it an effective lubricant in rotaxanes to enhance their shuttling.

## Introduction

Over the past decades, artificial molecular machines, or nanomachines, have proven extremely useful as devices that mimic macroscopic instruments.^1–6^ Rotaxanes,^7^ which consist of a dumbbell-shaped (or chain-like) molecule threaded onto a macrocycle, form the primary component of many types of nanomachines, like nanomuscles,^8–10^ nanovalves,^11–13^ nanorecorders,^14,15^ drug carriers,^16–18^ product lines,^19^ or locks.^20^ In these molecular architectures, the macrocyclic rings usually shuttle between different binding sites in response to an external stimulus,^21,22^ such as light,^23–26^ redox,^27,28^ a change of pH,^29,30^ temperature^31,32^ or solvent.^31,33^ The polarity of the solvent is generally considered to be crucial in controlling the motion of a rotaxane. However, in some hydrogen-bonded rotaxanes, like the amide-based rotaxane investigated by Panman *et al.*,^34^ only water can accelerate the shuttling rate of the macrocycle, whereas other polar solvents appear to have a limited effect. Why water alone possesses this unique property remains in large measure poorly understood. The main thrust of the present contribution is to shed light onto the distinctive role played by water in amide-based rotaxanes from the perspective of the thermodynamics of shuttling and the structural changes that accompany it.

Our previous theoretical investigations have revealed that contrary to chemical intuition, shuttling is usually coupled to other different movements. Mapping the free-energy landscape that describes shuttling, therefore, requires careful modeling of the underlying reaction coordinate.^35,36^ Toward this end, in the present study, we have decoupled the overall shuttling process of the amide-based rotaxane^34^ into several possible movements. We have determined the free-energy landscape characterizing the shuttling process in four different solvents, *i.e.* diethyl ether (Et_2_O), acetonitrile (ACN), ethanol (EtOH) and water. Key differences between the reaction pathways describing the shuttling of the macrocycle in water and in non-aqueous solvents rationalize the distinctive shuttling mechanism observed in water.

## Results and discussion

### Movements and reaction coordinate model

**Fig. 1:**
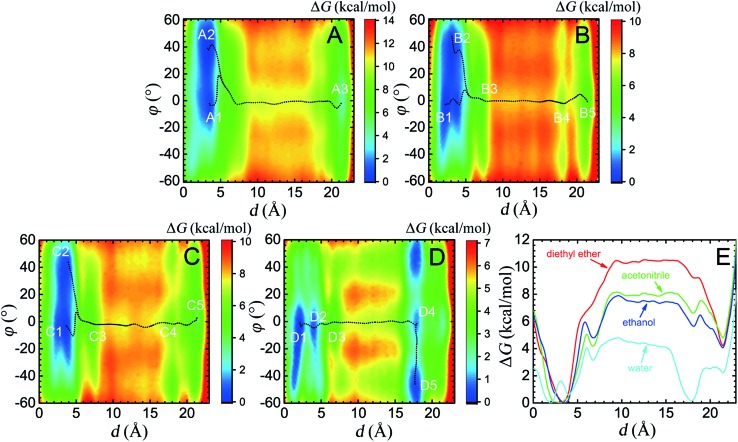
Free-energy calculations characterizing the translational movement and conformational change processes of the macrocycle. (A–D) Free-energy landscapes in diethyl ether, acetonitrile, ethanol and water, respectively, obtained by 4 × 2.2 μs simulations. White characters denote the local (meta)stable structures (local minima) of the rotaxane in different solutions. The black dotted lines indicate the least free energy pathways connecting the local minima. (E) Projection of the two-dimensional PMFs along the coarse variable *d*.

**Scheme 1:**
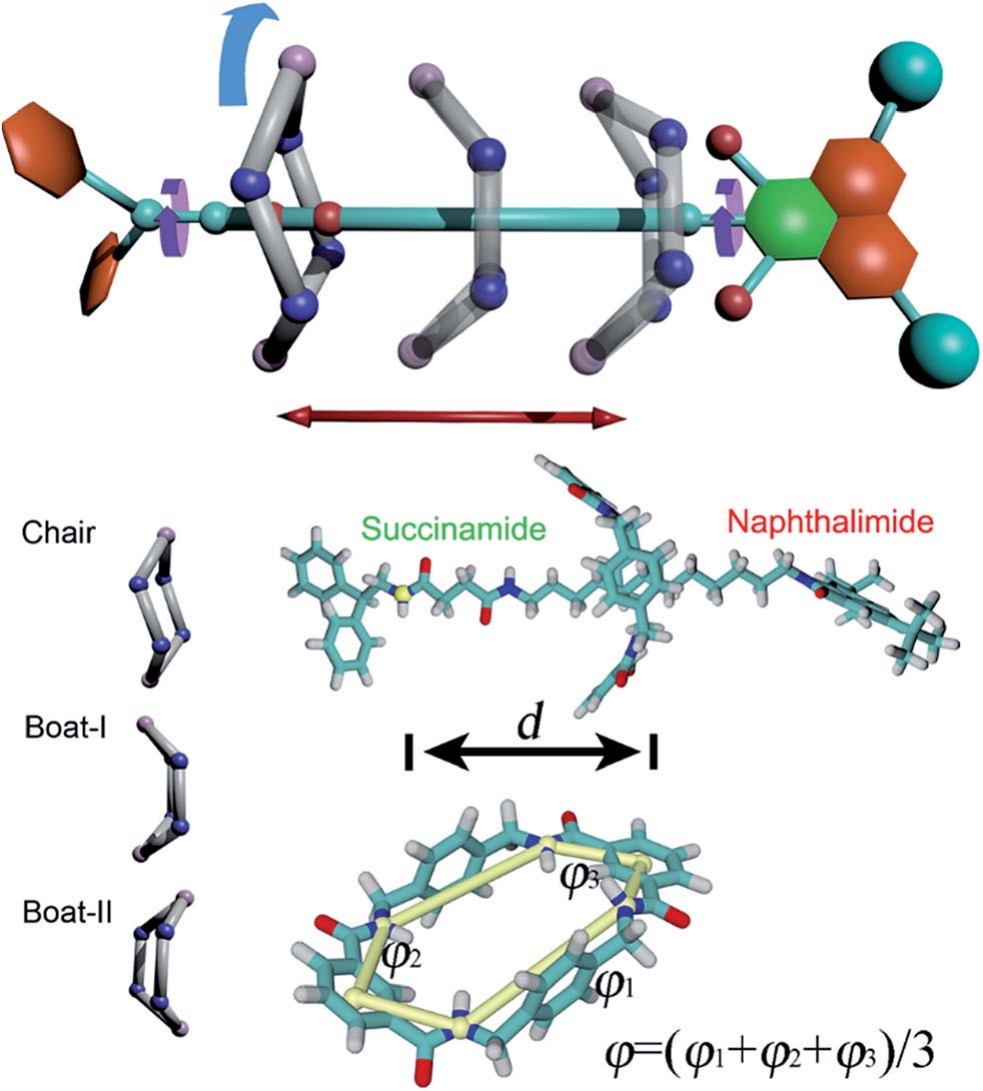
Upper panel, possible movements in the rotaxane. Red arrow, translation of the ring. Cyan arrow, rotation of the ring. Purple arrow, rotation of the stoppers. Lower left panel, isomerization of the macrocycle. Boat-I, -II: boat conformation facing to the left-hand and right-hand stoppers, respectively. Lower right panel, structure of the rotaxane and the coarse variables (*d*, *φ*) chosen to define the transition coordinate. *φ* = 0° denotes a boat-like conformation, while *φ* ≥ 20° or *φ* ≤ –20° indicates a chair-like conformation. Two binding sites on the thread are defined as “left-hand” and “right-hand”. The left-hand binding site is located at the succinamide group, while the right-hand one at the naphthalimide stopper.

The structure of the rotaxane investigated herein is depicted in Fig. 1A and S1 of the ESI.† The dumbbell-like molecule contains a succinamide moiety forming the left-hand binding site of the chain, and a naphthalimide group forming its right-hand binding site, capped with a stopper. The macrocycle is a benzylic amide ring including four amide moieties. In light of a preliminary simulation, the nature of the shuttling of this rotaxane was found to be particularly intricate. At least four possible movements were found to be coupled to each other, namely the translational movement, the conformational change and the rotation of the ring-like molecule, as well as the spin of the terminal groups of the dumbbell-shaped molecule (Scheme 1). Due to the inherent complexity of the intercoupled movements and the limitation of brute-force molecular dynamics simulations, importance-sampling-based free-energy calculations^37^ were employed here. Exhaustive scanning of the free-energy hyperplane is, however, computationally expensive when the number of dimension exceeds 3. We, therefore, dissected the shuttling process of the rotaxane carefully, and selected two primary coarse variables to form the transition coordinate, namely the distance, *d*, and the average dihedral angle, *φ*, defined in Scheme 1, to describe the translation and the conformational change of the ring-like molecule, respectively. To validate the choice of the coarse variable *φ*, the free-energy profile (or potential of mean force, PMF) for the isomerization of the macrocycle was determined, as shown in Fig. S2 of the ESI.† The resulting PMF clearly suggests that *φ* represents an appropriate variable. In the following two-dimensional free-energy calculations, the recently developed extended adaptive biasing force method^38–40^ was used. Alternate methodologies are detailed in the Methods section of the ESI.†

To support our choice of the reaction coordinate model, the PMF delineating the translation coupled with the conformational change of the ring in the rotaxane in a vacuum was calculated as depicted in Fig. S3.† Two binding sites can be clearly identified in the one dimensional free-energy profile of Fig. S3B,† namely, one on the succinamide group (left-hand binding site) and the other on the two carbonyl moieties of the naphthalimide moiety (right-hand binding site). The four types of intercoupled movements depicted in the Scheme 1 are thoroughly analyzed in Fig. S4–S6.† To further investigate whether the chosen transition coordinate is germane to characterize the movements of the rotaxane, or, in other words, whether the translation and the conformational change are the principal components of the rotaxane shuttling, a committor analysis^41,42^ was performed. The results shown in Fig. S7† clearly suggest that the applied coarse variables are suitable for modeling the rotaxane in motion.

### Effects of different solvents on the mechanism underlying shuttling

Four solvents with different properties were adopted in this study, *i.e.* diethyl ether, acetonitrile, ethanol and water. The properties of these solvents are highlighted in Table S1.† Detail of the molecular assemblies studied in this work is provided in Table S2.† The free-energy landscapes characterizing the translation and conformational change of the ring in different solvents are shown in Fig. 1A–D. The least free-energy pathways connecting the local minima on the hyperplane were determined using the LFEP algorithm.^43^

#### Diethyl ether

**Fig. 2:**
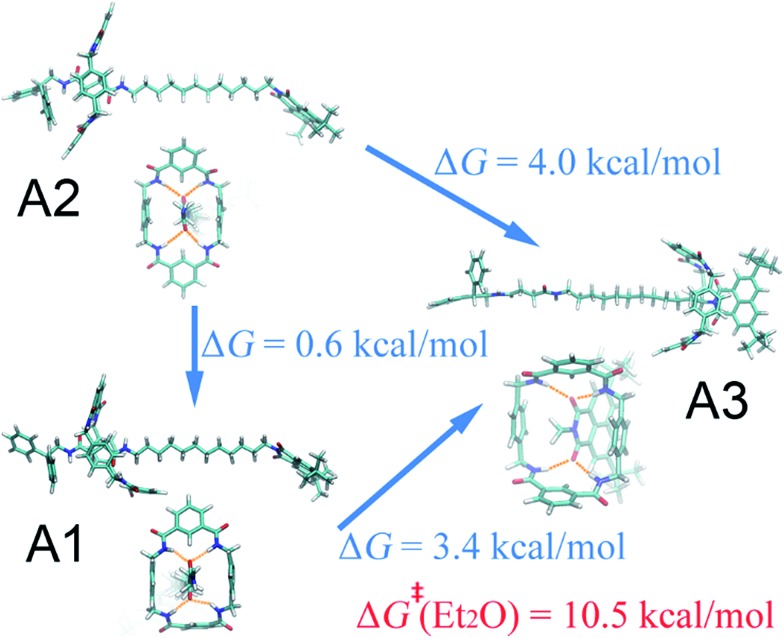
Representative three-dimensional arrangements of the rotaxane in diethyl ether. The free-energy differences between these structures are also shown. The activation free energy of the shuttling process is depicted in red.

As can be seen in Fig. 1A, in the non-polar diethyl ether, two local minima, A1 and A2, of nearly equal free energy, appear at *d* = 2–4 Å, where the ring-like molecule resides at the left-hand binding site, which is also visible in the PMF of Fig. 1E. The corresponding (meta)stable states are depicted in Fig. 2, wherein the macrocycle in A1 and A2 adopts a boat and a chair conformation, respectively. Hydrogen bonds are found to be formed between the amino groups of the macrocycle and the carbonyl groups of the chain. The macrocycle navigates between the two stable states until it translocates to the methylene chain, where it adopts the boat conformation. Only one stable three-dimensional arrangement, A3, emerges at *d* = 21–22 Å, where the ring is now located at the right-hand binding site. The corresponding structure is depicted in Fig. 2. Hydrogen bonds form between the amino groups of the macrocycle and the carbonyl groups of the naphthalimide moiety. As a result, the steric hindrance of the bulky stopper arising from the close contact between the latter two objects prevents the macrocycle from adopting a chair conformation. Breakdown of the one-dimensional free-energy change depicted in Fig. S8A† clarifies that intermolecular hydrogen bonding is the driving force responsible for shuttling in the non-polar solvent. The intermolecular hydrogen-bonding interaction is analyzed, as shown in Fig. S9A,† demonstrating that less hydrogen bonds are formed in structure A3 than in structure A1 and A2, thereby rationalizing the relative stability of the two binding sites observed in Fig. 1E. Further analysis of the conformational change reveals that during shuttling along the reaction path, the macrocycle experiences several times a boat–boat interconversion, as shown in Fig. S10.† Such isomerization happens in all four solvents.

#### Acetonitrile

**Fig. 3:**
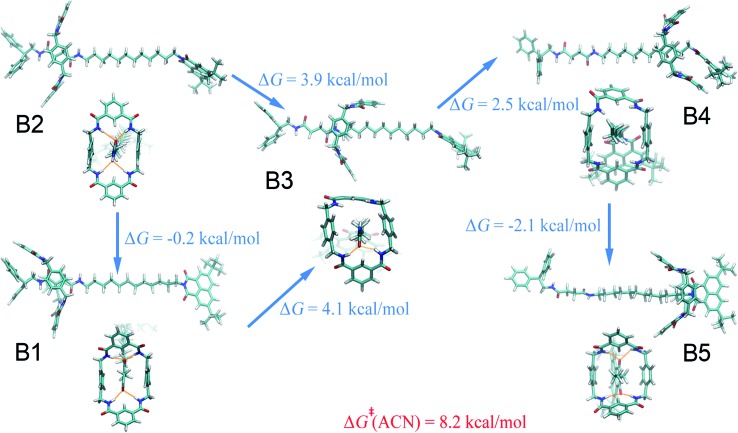
Representative spatial arrangements of the rotaxane in acetonitrile. The free-energy differences between the (meta)stable structures in acetonitrile and ethanol are shown. The activation free energies of the shuttling process are denoted in red.

Compared with the free-energy landscape characteristic of a non-polar solvent, two additional local minima emerge in the polar acetonitrile (B3 and B4 in Fig. 1B). Representative spatial arrangements of the rotaxane corresponding to the local minima in acetonitrile are shown in Fig. 3. Obviously, structures B1, B2 and B5 are akin to A1, A2 and A3, respectively, wherein hydrogen bonding contributes significantly to the stability of the three-dimensional arrangement. Both boat-I and boat-II orientations of the macrocycle exist in B1 and A1. In the boat-I arrangement, a phenylene group of the ring interacts with one phenyl moiety of the left stopper, and with the aliphatic chain instead in boat-II. In addition, the macrocycle was found to undergo two boat–boat isomerizations during shuttling—from B1 (boat-I) to B3 (boat-II), and a reverse transition, from B4 (boat-II) to B5 (boat-I). In spatial arrangement B3, only two of the four amino groups of the macrocycle can form hydrogen bonds with one carbonyl moiety of the succinamide group. This structure is stabilized by favorable solvophobic interactions of the aromatic groups of the ring with the aliphatic chain. In structure B4, no hydrogen bond is formed. Instead, the phenylene moiety of the benzylic amide ring interacts with the aromatic part of the terminal moiety through solvophobic π–π stacking. It can, therefore, be concluded that in acetonitrile, shuttling between B1 and B5 is primarily driven by hydrogen-bonding interactions, while solvophobic interactions also contributes to the intermediate states and the conformational changes. This result can also be reconciled with the free-energy decomposition reported in Fig. S8B and the hydrogen-bond statistics in Fig. S9B of the ESI.†

#### Ethanol

**Fig. 4:**
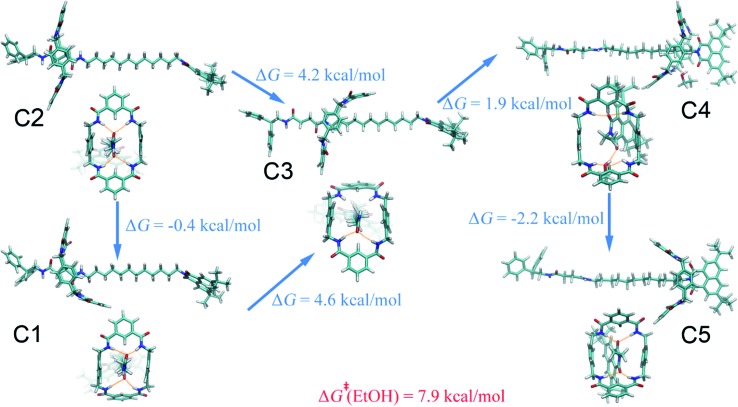
Representative structures of the rotaxane in ethanol. The free-energy differences between the (meta)stable motifs are shown. The activation free energies of the shuttling process are indicated in red.

The (meta)stable states of the amide-based rotaxane along the reaction pathway connecting the two binding sites in ethanol are similar to those in acetonitrile on account of the close polarities of the two solvents, as mirrored in the one- and two-dimensional free-energy landscapes in Fig. 1. The most striking difference appears in the metastable states B4 and C4, wherein the macrocyclic ring adopts the boat-I and boat-II conformation, respectively (see Fig. 4 and S10C†). This difference is due to the inherent properties of the two solvents, *i.e.*, acetonitrile is a hydrogen-bond acceptor, whereas ethanol is both an acceptor and a donor. As a result, the ethanol molecule bridges concomitantly a carbonyl moiety of the naphthalimide group and two amino groups of the macrocycle, stabilizing the three-dimensional arrangement C4. The driving force responsible for the shuttling of the ring, as can be proven by the free-energy decomposition in Fig. S8C of the ESI,† is also hydrogen-bonding interactions. The ability of ethanol to be a hydrogen donor cannot significantly reduce the free-energy barrier of shuttling, compared with acetonitrile.

#### Water

**Fig. 5:**
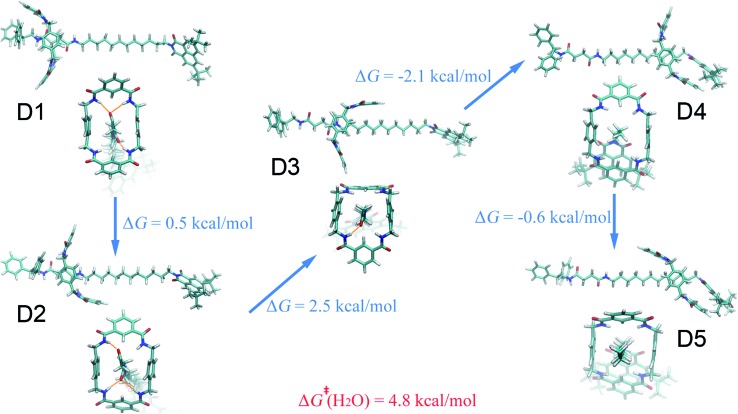
Representative structures of the rotaxane in aqueous solution. The free-energy differences between the distinct three-dimensional arrangements are shown. The activation free energies of the shuttling process are described in red.

The free-energy landscape characterizing the translation and isomerization of the macrocycle in water differs significantly from those determined in diethyl ether, acetonitrile and ethanol (Fig. 1D and E). In particular, in Fig. 1E, compared with the PMF characteristic of non-aqueous solvents, the free-energy barrier against shuttling of the rotaxane is much lower, which is in line with the idea of “lubrication effect” by water, as has been described in experiments.^36^ In addition, both the left- and right-hand binding sites shift leftward compared to other solvents. Further structural analysis reveals a different conformational change for the ring during shuttling, compared with non-aqueous environments, as illuminated in Fig. 5. At the binding site of the succinamide moiety, the macrocycle adopts a boat-I conformation (D1). The boat-I conformation of the ring is no longer an energetically favorable state when the ring starts to move, and, in stark contrast, the boat-II conformation becomes dominant (D2). The macrocycle remains in the boat-II conformation when it moves along the aliphatic chain, until it reaches the metastable state D4 at the right-hand binding site. Then, the macrocycle isomerizes quickly to a chair conformation, and adopts the stable three-dimensional arrangement D5. In D5, the amino groups of the benzylic amide ring no longer bind to the carbonyl groups of the naphthalimide moiety, as evinced in Fig. 5.

The special property of the rotaxane in water can be intuitively explained by the representative structures depicted in Fig. 5. Hydrogen-bonding interactions of the macrocycle with the chain are significantly weakened in water, as shown in Fig. S9.† In motifs D1 and D2, the phenylene groups of the ring interact with the stopper and/or the aliphatic chain connecting with the succinamide moiety. The macrocycle does not interconvert spontaneously in a chair conformation at the left-hand binding site (Fig. S10†) to minimize the surface area of the hydrophobic part of the chain exposed to the aqueous environment (Fig. S11†). No ring-chain hydrogen bond is formed in structures D4 and D5 (Fig. 5 and S9†). A favorable π–π stacking interaction greatly stabilizes structure D5 instead, making the latter almost as energetically favorable as structure D1. It can be inferred that hydrogen bonding is no longer the driving force responsible for the translocation of the macrocycle from the left-hand binding site to the right-hand one. On the contrary, hydrophobic interactions contribute the most to the shuttling process. This result is also supported by the free-energy decomposition of Fig. S8D.†

### Shuttling rate and mechanism of lubrication by water

**Fig. 6:**
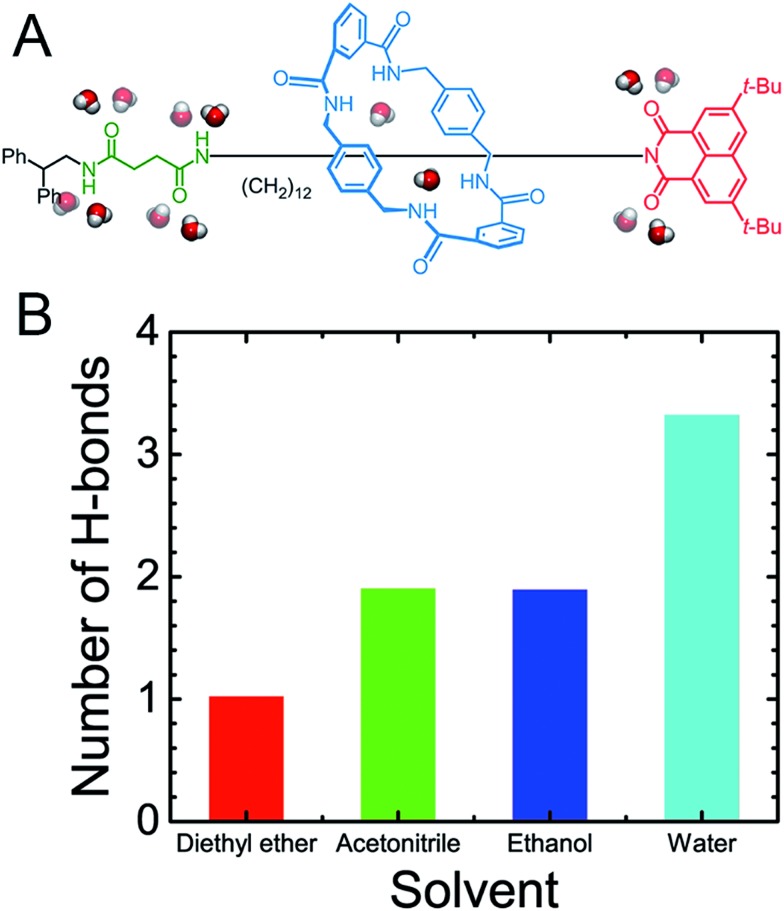
(A) Water stabilizes the amides of the macrocyclic ring and the chain-like molecule. (B) The average number of hydrogen bonds between the amino groups of the ring and the oxygen/nitrogen atom of the solvents when the coarse variable *d* = 12–16 Å, *i.e.* the ring resides over the aliphatic part of the chain.

As may be seen in Fig. 1E, the free-energy barrier to be overcome for translocating the ring from the left-hand binding site to the right-hand one drops as the polarity of the solvent increases. In particular, the free-energy barrier reduced sharply in water, compared with non-aqueous solvents, thereby, accelerating shuttling significantly. The inherent properties of water can rationalize its unique lubricant role. First, when the macrocycle interacts with the succinamide moiety, the contribution to the free energy of the hydrogen bonds formed between the ring and the chain is greatly reduced due to the high polarity of water. The pronounced disrupting effect of water on the ring-chain hydrogen bonds can be seen in Fig. S9 (ESI†). Hydrophobic interactions, therefore, become dominant and drive the shuttling of the macrocycle ring from left to right. Second, the phenylene groups of the macrocycle can interact with the hydrophobic part of the supramolecular assembly, either the aliphatic chain or the aromatic stopper, during shuttling. This solvophobic interaction is significantly stronger in water, compared with other solvents. Last, when the ring moves away from the succinamide moiety to the alkly chain, water molecules can form hydrogen bonds with the carbonyl groups of the chain and the amino groups of the ring (Fig. 6), thus, compensating the energy loss due to breaking hydrogen bonds between the ring and the chain. As a result, the free-energy barrier against translocating the ring from the left-hand binding site to the right-hand one greatly reduced. Diethyl ether and acetonitrile, in stark contrast, are not hydrogen bond donors and, hence, cannot form hydrogen bonds with the carbonyl moieties of the chain. Moreover, diethyl ether, acetonitrile and ethanol are larger in volume, compared with the water molecule. It is, therefore, entropically difficult for the former molecules to insert into the cavity formed by the chain and the macrocycle to stabilize the amino moieties of the latter, as indicated in Fig. 6. To prove the proposed volumetric effect, the hydrogen bonds formed between the ring and the solvents when the coarse variable *d* = 12–16 Å, *i.e.*, the ring is around the aliphatic part of the chain, were analyzed. Fig. 6 clearly indicates that the number of such hydrogen bonds in water is much greater than in any other solvent, thus, suggesting the importance of the small volume of water in acting as a lubricant.

## Conclusions

The amide ring of the rotaxane investigated in this study adopts different conformations along the reaction pathways connecting the left-hand and right-hand binding sites in the four solvents examined in this study on account of their distinct properties. The driving forces responsible for the shuttling of the macrocycle directly contribute to the distinct three-dimensional arrangements of the rotaxane. In diethyl ether, acetonitrile and ethanol, the translation coupled with conformational transition is driven by hydrogen bonding. In stark contrast, the left-to-right shuttling in an aqueous solution is controlled chiefly by hydrophobic interactions. The low free-energy barrier against shuttling stems from lubrication effect by water. The special properties of water can be summarized as follows—a high polarity, the ability of being both a hydrogen donor and an acceptor, and a very small molecular volume, which can greatly weaken the hydrogen bonds between the ring and the dumbbell-like molecule and stabilize the transition states. These properties of water, hence, greatly reduce the friction between the chain and the ring of the rotaxane due to hydrogen bonding, making it act as a lubricant to enhance the shuttling in this supramolecular architecture. This conclusion obtained from amide-based rotaxanes can be used to rationalize the lubricating role of water in the motion of biological and abiological machines consisting of hydrogen-bonded components.^44,45^ For example, the rotation of the motor protein ATPase is generally believed to be controlled by electrostatic interactions. Water can, however, help hydrophobic residues induce local deformations prior to electrostatically driven rotation, thereby reducing the barriers of side-chain dissociation and association that drive stalk rotation.^45^

Taking advantage of the carefully chosen transition coordinate and subsequent free-energy calculations, as well as other analyses, we bring to light the intertwined movements of the rotaxane, which lays the groundwork for exploring the features of more complex molecular machines. Moreover, we emphasize that translation coupled with conformational change of the rotaxane may be completely altered by solvation in a different environment, thereby providing a new perspective for the rational design of solvent-controlled nanomachines. Among the most common solvents ordinarily found at the bench, water is in many respects particularly unique, as has been shown in this study. Its special properties may modulate the driving force of molecular architectures, leading to unexpected phenomena.
