# Supplementary material for: The lubricating role of water in the shuttling of rotaxanes
Source: Chem Sci. 2017 May 16;8(7):5087–94. doi: 10.1039/c7sc01593c (PMC5613241; doi:10.1039/c7sc01593c)
Supplement: Supplementary file 1 [file SC-008-C7SC01593C-s001.pdf]

Electronic Supplementary Material (ESI) for Chemical Science.
This journal is © The Royal Society of Chemistry 2017

Electronic Supplementary Information for

The Lubricating Role of Water in the Shuttling of Rotaxanes

Haohao Fu,[a] Xueguang Shao,[a,b,c] Christophe Chipot,[d,e,f] and Wensheng Cai*[,a,b]

[a]Research Center for Analytical Sciences, College of Chemistry, Tianjin Key Laboratory of Biosensing and Molecular Recognition, Nankai University, Tianjin 300071, China

[b]Collaborative Innovation Center of Chemical Science and Engineering (Tianjin), Tianjin 300071, China

[c]State Key Laboratory of Medicinal Chemical Biology (Nankai University), Tianjin 300071, China

[d]Laboratoire International Associé Centre National de la Recherche Scientifique et University of Illinois at Urbana-Champaign, Unité Mixte de Recherche No. 7565, Université de Lorraine, B.P. 70239, 54506 Vandœuvre-lès-Nancy cedex, France

[e]Theoretical and Computational Biophysics Group, Beckman Institute, University of Illinois at Urbana-Champaign, Urbana, Illinois 61801

[f]Department of Physics, University of Illinois at Urbana−Champaign, 1110 West Green Street, Urbana, Illinois 61801, United States

*E-mail: wscai@nankai.edu.cn.

Table S1. The properties of the solvents used in this study.[a]

Solvents	Relative polarity	H-bond donor or acceptor
Diethyl ether	0.117	Acceptor
Acetonitrile	0.460	Acceptor
Ethanol	0.654	Both
Water	1.000	Both

[a]From Reichardt, C.; Welton, T. Solvents and solvent effects in organic chemistry. John Wiley & Sons: 2011.

Table S2. Detail of the molecular assemblies examined in this study.

Solvent	Number of atoms	Number of solvent molecules	Size of the simulation box ($Å^3$)[a]	Simulation time (μs)
Vacuum	190	-	-	2.2
Diethyl ether	10570	692	45×45×66	2.2
Acetonitrile	9064	1479	47×46×70	2.2
Ethanol	13267	1453	48×48×70	2.2
Water	11437	3749	45×44×66	2.2

[a]The size of the solvent boxes guarantees a minimum distance of 15 Å from any atom of the rotaxane to any edge of the simulation box.

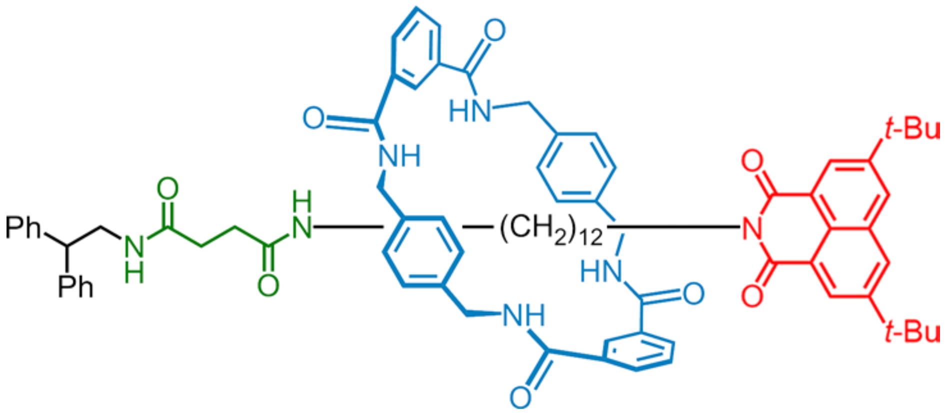

Figure S1. Structure of the rotaxane investigated in this study. Green, succinamide moiety; red, naphthalimide group, and cyan, macrocyclic ring.

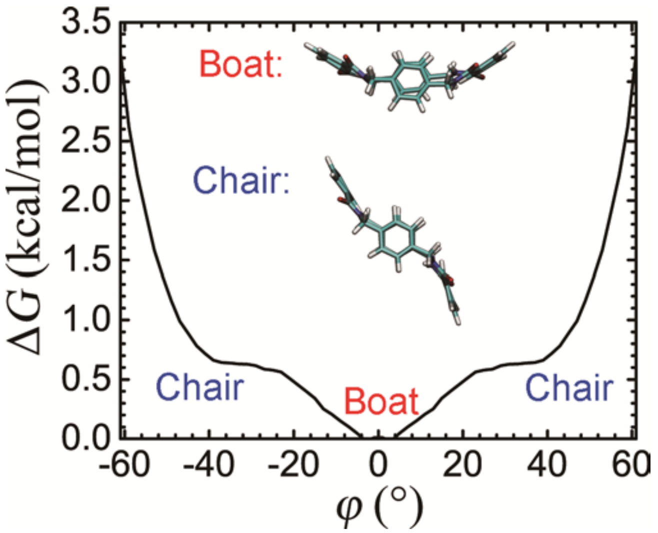

Figure S2. Free-energy calculation characterizing the isomerization of the ring-like molecule of the rotaxane in this study. The combination of three dihedral angles shown in Figure 1 was used as the transition coordinate. This Figure clearly suggests that the model reaction coordinate can be used to describe the conformational change of the ring. Moreover, the free-energy difference between the boat and the chair motifs is as less as $k_B T$ (~0.6 kcal/mol).

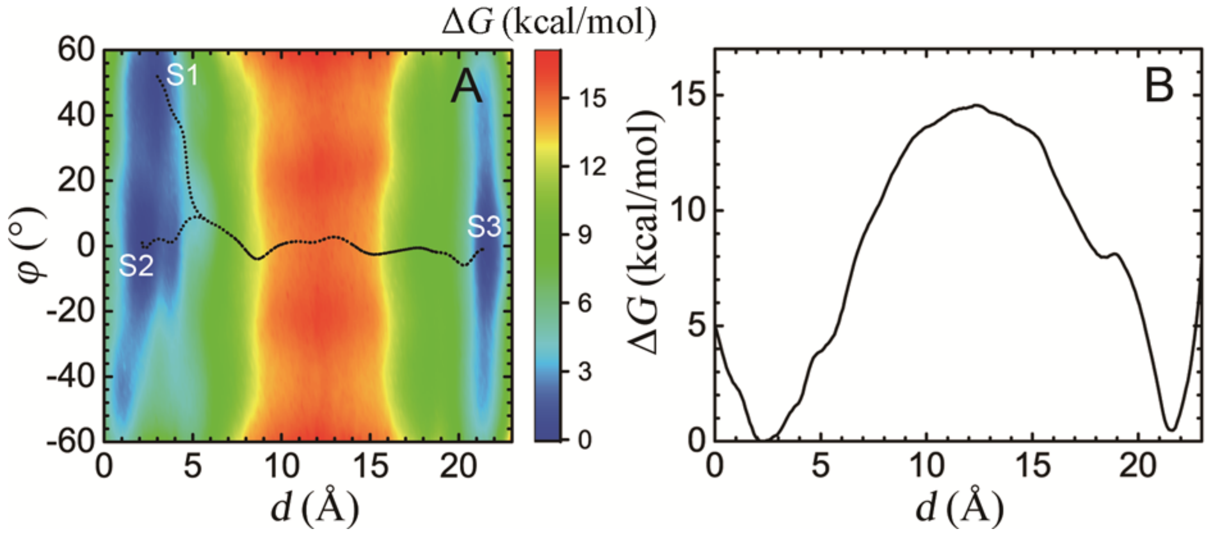

Figure S3. (A) Free-energy landscape characterizing the shuttling process of the rotaxane in vacuum, obtained by a 2.2 μs simulation. White characters denote the typical structures of the rotaxane. The black dotted lines indicate the least free energy pathways connecting stable states S1 – S3 and S2 – S3. (B) Projection of the two-dimensional PMF along the coarse variable *d*.

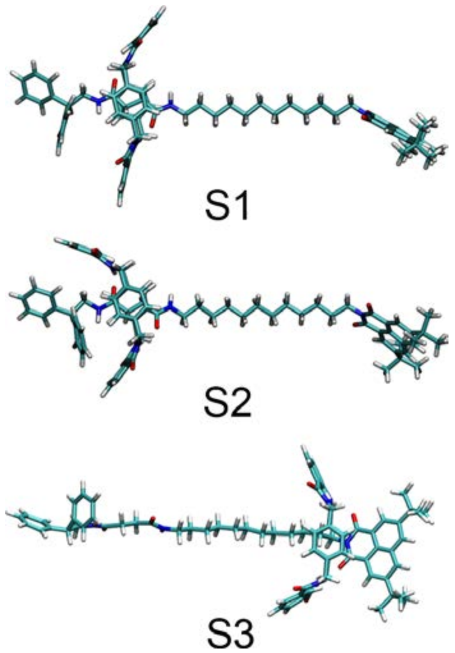

Figure S4. Conformational changes of the ring during its shuttling process. Representative three-dimensional arrangements of the rotaxane in vacuum.

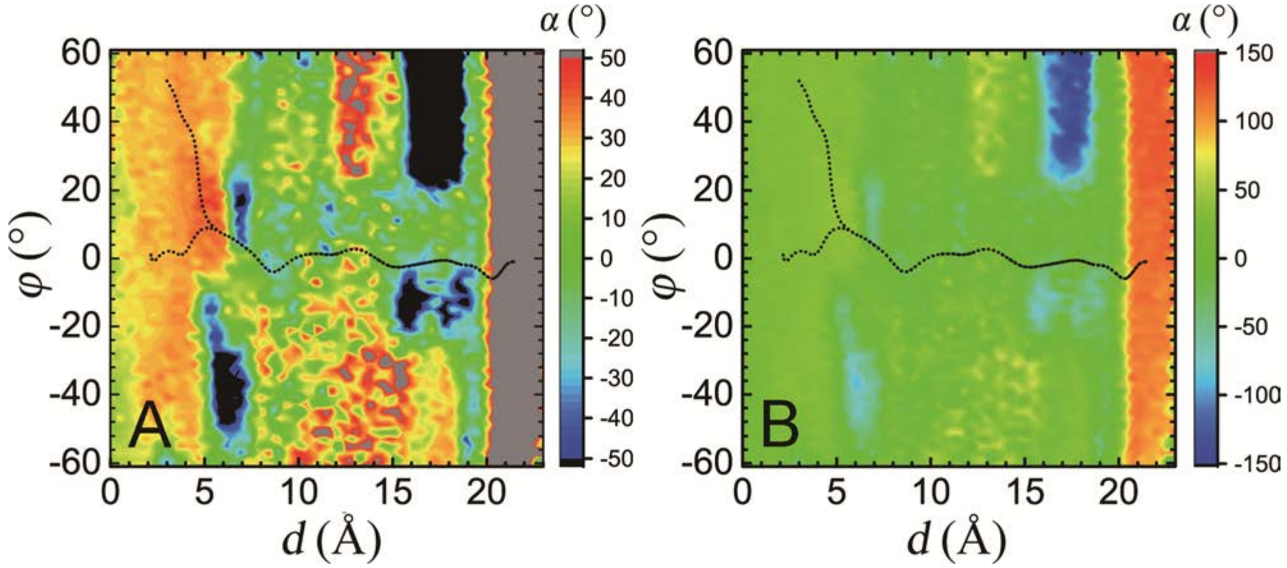

Figure S5. Rotation of the ring of the rotaxane coupled with its translation and isomerization. The average angles between the connection of the two phenylene of the ring-like molecule and the x-axis were measured in vacuum along the transition coordinate. The least free energy pathway is indicated by the black dotted lines. (A) and (B) show the same result in different color scales. The ring rotates from -60° to 130° during the shuttling.

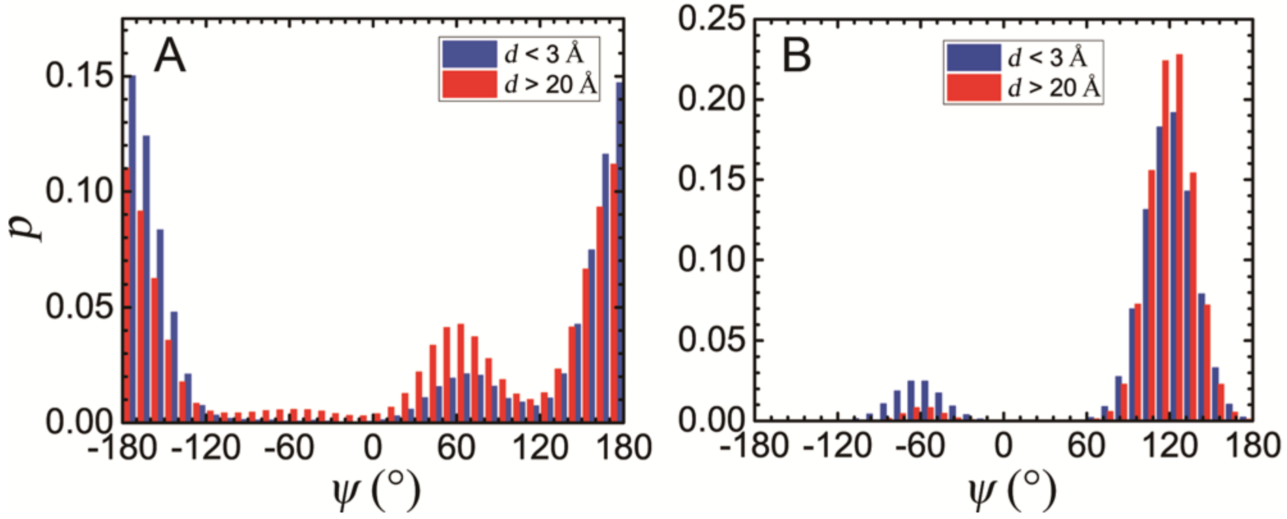

Figure S6. Rotation of the terminal groups of the dumbbell-like molecule coupled with the shuttling of the rotaxane. The angles between the connection of the two phenyls of the left stopper (A) and two oxygen atoms of the naphthalimide group (B) and the x-axis were measured. The distributions of the angles are calculated when the ring is at the left-hand side (d < 3 Å) and at the right-hand side (d > 20 Å). The $(C_6H_5)_2CH$- can rotate from ψ = 60° to ψ = ±180°. The rotation becomes more free when the ring is at the right-hand side. The results of the naphthalimide moiety are similar to those of the left stopper.

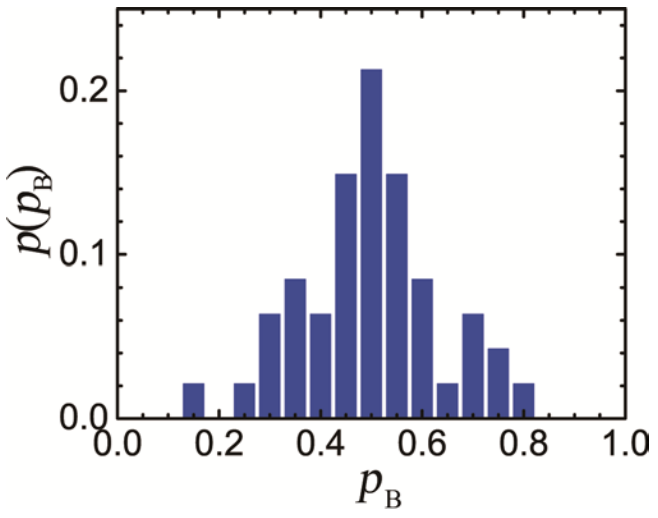

Figure S7. Distribution of the committor, p_B, at the positions near the saddle points around d = 12.3 Å, φ= 1° in the free-energy surface shown in Figure S3. The distributions are Gaussian-like with a peak at p_B = 0.5, suggesting the chosen coarse variables be suitable for studying the movement of the rotaxane.

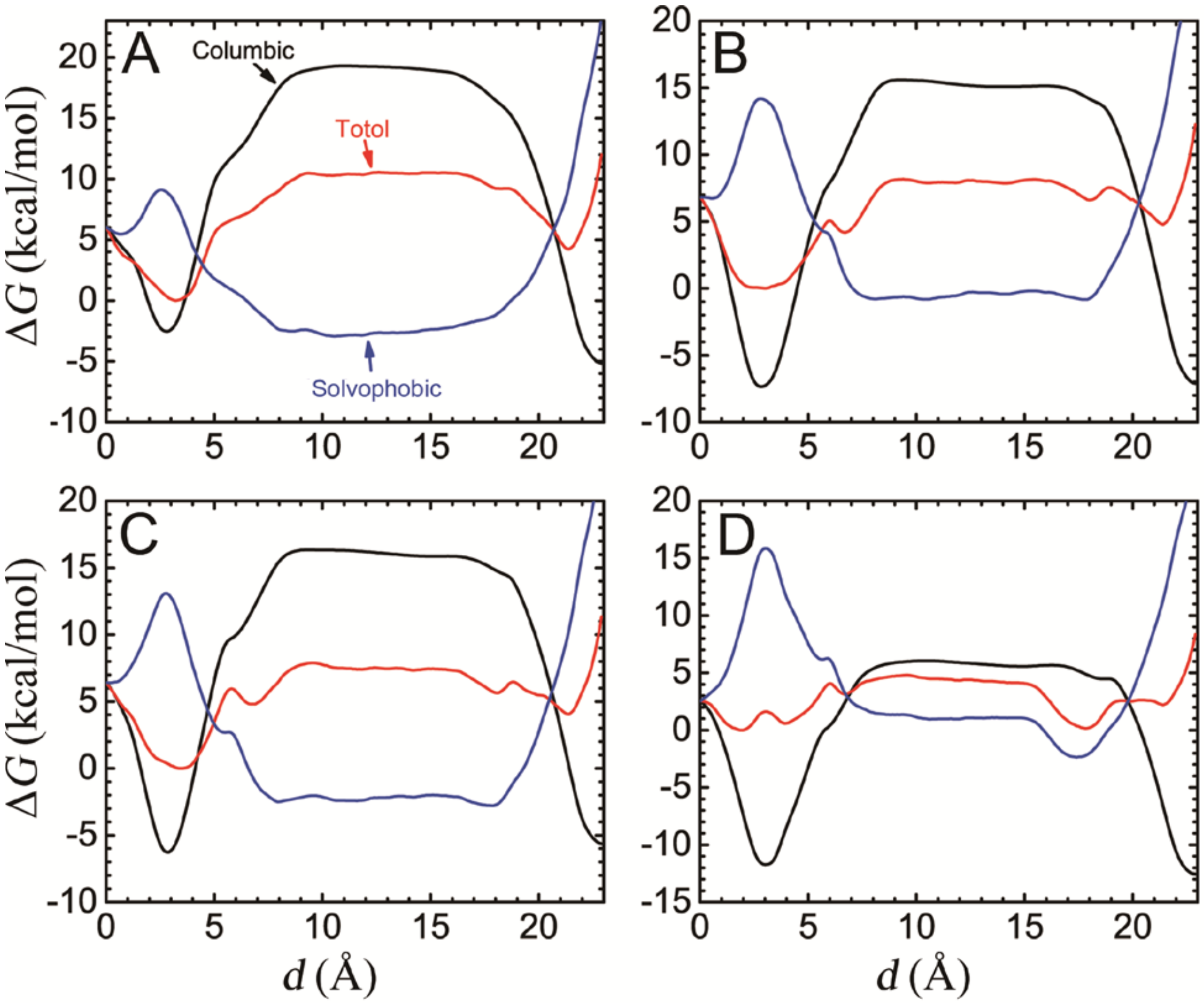

Figure S8. One dimensional free-energy decomposition for the PMFs describing the shuttling of the rotaxane in (A) diethyl ether, (B) acetonitrile, (C) ethanol and (D) water. The dumbbell-ring columbic interaction mainly mirrors the contribution of hydrogen bonds while the solvophobic interaction includes the contributions of dumbbell-macrocycle van der Waals force and the free energy of dissolution of the rotaxane.

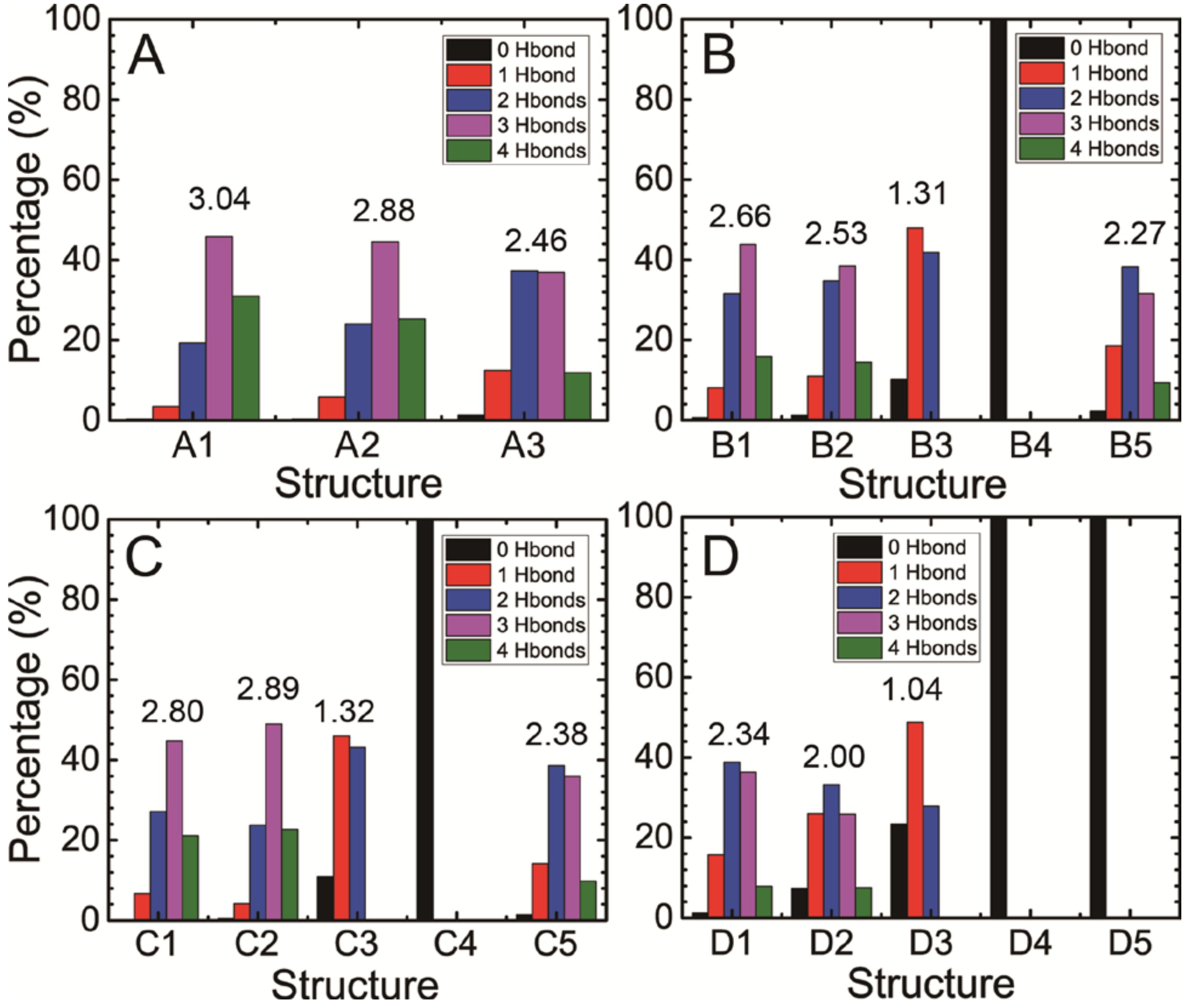

Figure S9. Distribution of the hydrogen bonds formed between the macrocycle and the thread or stopper of the typical structures, (A) in diethyl ether, (B) in acetonitrile, (C) in ethanol and (D) in water, labeled in Figure 1A-D. The black numbers denote the average numbers of hydrogen bonds in each stable structure.

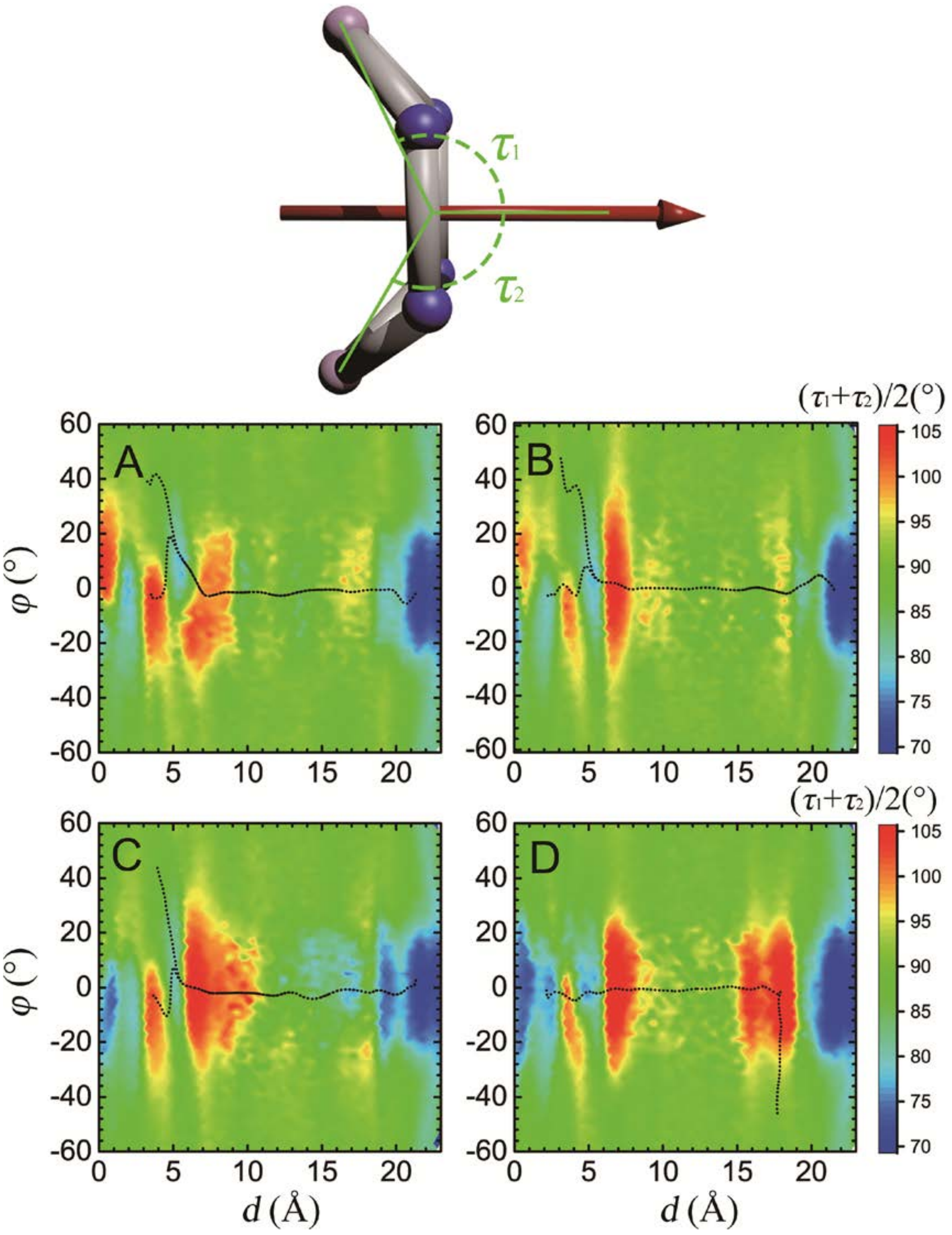

Figure S10. Boat-boat transformation of the macrocycle in (A) diethyl ether, (B) acetonitrile, (C) ethanol and (D) water. Upper panel, the definition of the angle τ_1 and τ_2. $(\tau_1 + \tau_2) / 2 < 85°$ implies that the ring shows a boat conformation, with the two phenylene groups directing toward the left (Boat-I in Figure 1). $(\tau_1 + \tau_2) / 2 > 95°$ suggests a boat conformation, with the two phenylene groups directing toward the right (Boat-II in Figure 1). $85° < (\tau_1 + \tau_2) / 2 < 95°$ represents a chair or a boat conformation with an arbitrary orientation.

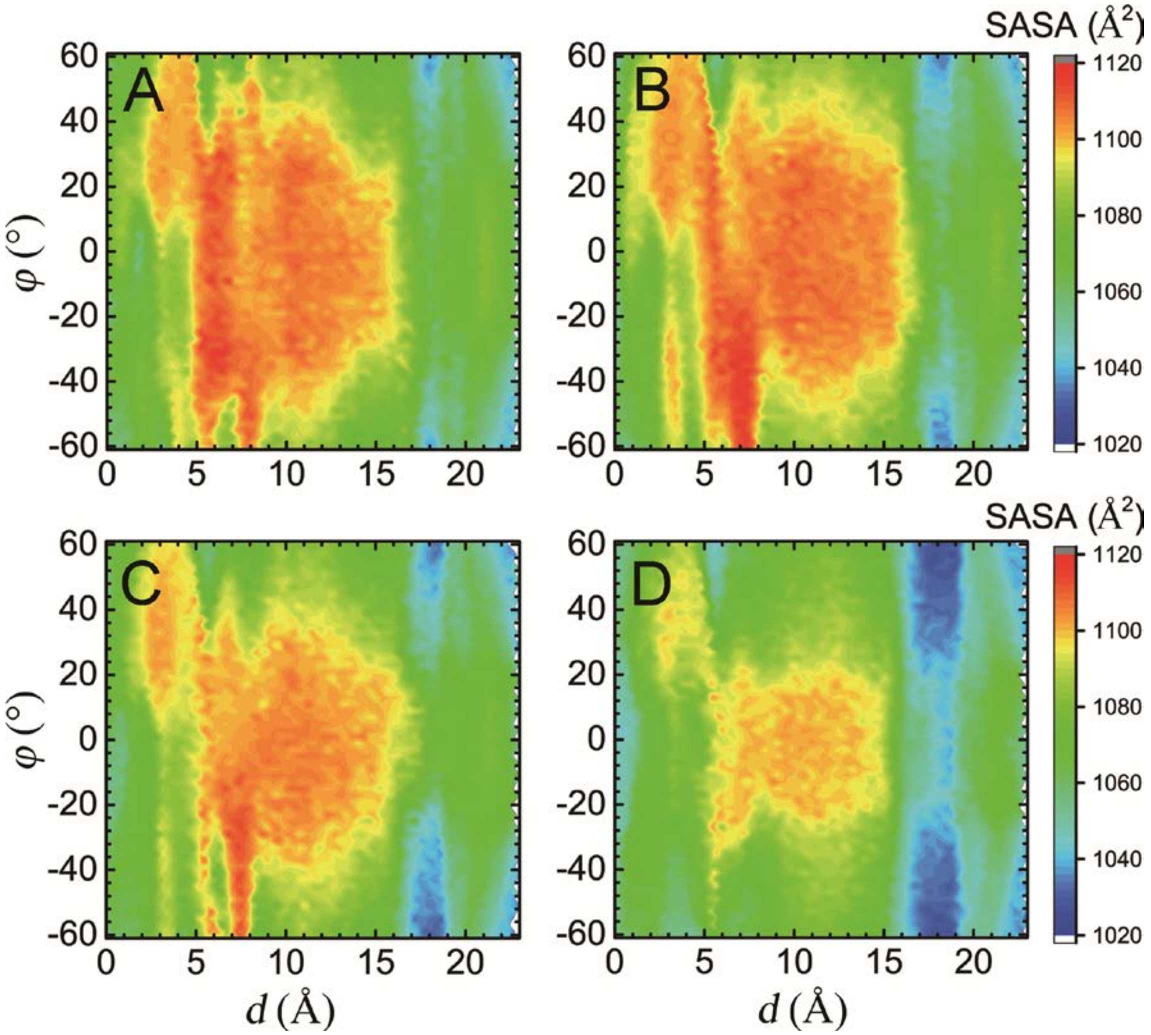

Figure S11. Solvent-accessible surface area (SASA) of the chain-like molecule along the transition coordinate in (A) diethyl ether, (B) acetonitrile, (C) ethanol and (D) water, respectively. When the ring bind to the left site, the chair conformation will lead to an exposure of the chain-like molecule to the solvent (See for example, d = ~3.5 Å, φ= ~40°), which makes this three-dimensional arrangement unfavorable in water. On the contrast, structure D5 (corresponding to d = 17.7 Å, φ= ±47°) is extremely favorable in hydrophobic interaction.

Methods

Molecular dynamics simulations. All the atomistic MD simulations presented herein were performed using the parallel, scalable program NAMD 2.11.[1] Water was described by the TIP3P model[2] while other molecules in this study were modeled by the CHARMM General Force Field (CGenFF).[3] The temperature and the pressure were maintained at 300 K and 1 atm, respectively, employing Langevin dynamics and the Langevin piston method.[4] Chemical bonds involving hydrogen atoms were constrained to their experimental lengths by means of the SHAKE/RATTLE[5-6] and SETTLE algorithms.[7] The r-RESPA multiple-time-stepping algorithm[8] was applied to integrate the equations of motion with a time step of 2 and 4 fs for short- and long-range interactions, respectively. A smoothed 12 A spherical cutoff was applied to truncate van der Waals and short-range electrostatic interactions. Periodic boundary conditions (PBCs) were applied in the three directions of Cartesian space. Long-range electrostatic forces were taken into account by the particle-mesh Ewald scheme.[9] Visualization and analysis of the MD trajectories were performed with VMD 1.9.2.[10]

Free-energy calculations. The free-energy calculations reported herein were carried out utilizing the multiple-walker extended adaptive biasing force (MW-eABF) algorithm.[11-13] To increase the efficiency of the calculations, the free-energy surface was broken down into six consecutive, non-overlapping windows. Instantaneous values of the force were accrued in bins, with 0.1 Å × 2° wide. The sampling time required to determine each PMF was 2.2 µs. The least free-energy pathway connecting the minima of the two-dimensional free-energy landscapes was located using the LFEP algorithm.[14] The concept of committor[15,16] was utilized to demonstrate that the model reaction coordinates formed by collective variables d and φ corresponds to an appropriate choice.

Assessment of transition coordinates using a committor analysis. For the least free-energy pathway connecting stable state A (d = 2.15 Å, φ = 1°) to stable state B (d = 21.55 Å, φ = -1°), 100 different structures at the position (the saddle point) around d = 12.3 Å, φ = 1° in the free-energy surface were extracted from the trajectories generated in the eABF simulations (see

Figure S3). For each structure, 100 5000-step equilibrium simulations were carried out with different initial velocities. The frequency characterizing the molecular assembly tending to relax to state B before reaching state A, p_B, was calculated for each structure. The distribution of p_B for the 100 distinct structures is provided in Fig. S7. This distribution is Gaussian-like, with a peak at p_B = 0.5, which suggests that the chosen coarse variables are suitable for studying the movement of the macrocycle in the rotaxane.

Comparison between classical ABF and eABF

In the classical ABF method, the biasing force is added to the groups of atoms at play, whereas in extended ABF, the bias is applied onto a fictitious particle coupled to the coarse variable of interest by means of a stiff spring. In most cases, classical ABF is appropriate for multidimensional free-energy calculations in the limit of low dimensionality problems — typically $n \leq 3$. However, extended ABF must be employed in the following cases,

i) The second derivative of the coarse variable is not available in the free-energy calculation engine.

ii) The chosen coarse variables are not independent from each other.

iii) The chosen coarse variables are coupled to geometric restraints or holonomic constraints.

In this study, the variable describing the conformational change of the macrocycle, $\varphi=(\varphi_1+\varphi_2+\varphi_3)/3$, consists of three coarse variables coupled to each other. Extended ABF must, therefore, be used in the free-energy calculations.

Moreover, the eABF method possesses also a much higher convergence rate compared with the original algorithm. See ref. 12 and 17 for more information.

Lubrication effect by water on the motion of abiological and biological molecular machines

Water can greatly weaken hydrogen bonds and stabilize transition states due to its high polarity, ability to act as both a hydrogen donor and acceptor, and very small molecular volume. Lubrication by water, therefore, is universal for all the hydrogen-bonding driven molecular

machines, including the wheel-and-axle machine reported by Panman et al.[18] The ability of changing the driving force from hydrogen bonding to hydrophobic interaction is, however, specific for those complexes possessing large hydrophobic groups. The wheel-and-axle machine only features succinamide moieties as stoppers, which are not large enough to help water convert the driving force from hydrogen bonding to hydrophobic interaction, as can be inferred from structure D1 and D2 in Fig. 5.

In addition to its role in abiological, artificially designed molecular machines, water also plays a key role in biological machines. For example, the rotation of the motor protein ATPase is generally believed to be controlled by electrostatic interactions. Water can, however, help hydrophobic residues induce local deformations prior to electrostatically driven rotation, thereby reducing the barriers of side-chain dissociation and association that drive stalk rotation.[19]

References

1 J. C. Phillips, R. Braun, W. Wang, J. Gumbart, E. Tajkhorshid, E. Villa, C. Chipot, R. D. Skeel, L. Kale, K. Schulten, Scalable molecular dynamics with NAMD. *J. Comput. Chem.* 2005, **26**, 1781-1802.

2 W. L. Jorgensen, J. Chandrasekhar, J. D. Madura, R. W. Impey, M. L. Klein, Comparison of simple potential functions for simulating liquid water. *J. Chem. Phys.* 1983, **79**, 926-935.

3 K. Vanommeslaeghe, E. Hatcher, C. Acharya, S. Kundu, S. Zhong, J. Shim, E. Darian, O. Guvench, P. Lopes, I. Vorobyov, CHARMM general force field: A force field for drug‐like molecules compatible with the CHARMM all‐atom additive biological force fields. *J. Comput. Chem.* 2010, **31**, 671-690.

4 S. E. Feller, Y. Zhang, R. W. Pastor, B. R. Brooks, Constant pressure molecular dynamics simulation: the Langevin piston method. *J. Chem. Phys.* 1995, **103**, 4613-4621.

5 H. C. Andersen, RATTLE: A "Velocity" version of the SHAKE algorithm for molecular dynamics calculations. *J. Comput. Phys.* 1983, **52**, 24-34.

6 J.-P. Ryckaert, G. Ciccotti, H. J. Berendsen, Numerical integration of the cartesian equations

of motion of a system with constraints: molecular dynamics of n-alkanes. *J. Comput. Phys.* 1977, **23**, 327-341.

7 S. Miyamoto, P. A. Kollman, SETTLE: an analytical version of the SHAKE and RATTLE algorithm for rigid water models. *J. Comput. Chem.* 1992, **13**, 952-962.

8 M. Tuckerman, B. J. Berne, G. J. Martyna, Reversible multiple time scale molecular dynamics. *J. Chem. Phys*. 1992, **97**, 1990-2001.

9 T. Darden, D. York, L. Pedersen, Particle mesh Ewald: An N· log (N) method for Ewald sums in large systems. *J. Chem. Phys.* 1993, **98**, 10089-10092.

10 W. Humphrey, A. Dalke, K. Schulten, VMD: visual molecular dynamics. *J Mol Graphics* 1996, **14**, 33-38.

11 J. Comer, J. C. Phillips, K. Schulten, C. Chipot, Multiple-replica strategies for free-energy calculations in NAMD: Multiple-walker adaptive biasing force and walker selection rules. *J. Chem. Theory Comput.* 2014, **10**, 5276-5285.

12 H. Fu, X. Shao, C. Chipot, W. Cai, Extended adaptive biasing force algorithm. An on-the-fly implementation for accurate free-energy calculations. *J. Chem. Theory Comput.* 2016, **12**, 3506-3513.

13 J. Comer, J. C. Gumbart, J. Hénin, T. Lelièvre, A. Pohorille, C. Chipot, The adaptive biasing force method: everything you always wanted to know but were afraid to ask. *J. Phys. Chem. B* 2014, **119**, 1129-1151.

14 B. Ensing, A. Laio, M. Parrinello, M. L. Klein, A recipe for the computation of the free energy barrier and the lowest free energy path of concerted reactions. *J. Phys. Chem. B* 2005, **109**, 6676-6687.

15 P. G. Bolhuis, D. Chandler, C. Dellago, P. L. Geissler, Transition path sampling: Throwing ropes over rough mountain passes, in the dark. *Annu. Rev. Phys. Chem.* 2002, **53**, 291-318.

16 A. C. Pan, D. Sezer, B. Roux, Finding transition pathways using the string method with swarms of trajectories. *J. Phys. Chem. B* 2008, **112**, 3432-3440.

17 A. Lesage, T. Lelièvre, G. Stoltz, J. Hénin, Smoothed biasing forces yield unbiased free

energies with the extended-system adaptive biasing force method. *J. Phys. Chem. B* 2017, **121**, 3676–3685.

18 M. R. Panman, B. H. Bakker, D. den Uyl, E. R. Kay, D. A. Leigh, W. J. Buma, A. M. Brouwer, J. A. Geenevasen, S. Woutersen, Water lubricates hydrogen-bonded molecular machines. *Nat. Chem.* 2013, **5**, 929-934.

19 A. Singharoy, C. Chipot, M. Moradi, K. Schulten, Chemomechanical coupling in hexameric protein–protein interfaces harness energy within V–type ATPases. *J. Am. Chem. Soc.* 2017, **139**, 293–310.
